# Predicting Prolonged Length of Hospital Stay for Peritoneal Dialysis–Treated Patients Using Stacked Generalization: Model Development and Validation Study

**DOI:** 10.2196/17886

**Published:** 2021-05-19

**Authors:** Guilan Kong, Jingyi Wu, Hong Chu, Chao Yang, Yu Lin, Ke Lin, Ying Shi, Haibo Wang, Luxia Zhang

**Affiliations:** 1 National Institute of Health Data Science Peking University Beijing China; 2 Advanced Institute of Information Technology Peking University Hangzhou China; 3 Renal Division, Department of Medicine Peking University First Hospital Peking University Institute of Nephrology Beijing China; 4 Department of Medicine and Therapeutics LKS Institute of Health Science The Chinese University of Hong Kong Hong Kong China; 5 China Standard Medical Information Research Center Shenzhen China; 6 Clinical Trial Unit First Affiliated Hospital of Sun Yat-Sen University Guangzhou China

**Keywords:** peritoneal dialysis, prolonged length of stay, machine learning, prediction model, clinical decision support

## Abstract

**Background:**

The increasing number of patients treated with peritoneal dialysis (PD) and their consistently high rate of hospital admissions have placed a large burden on the health care system. Early clinical interventions and optimal management of patients at a high risk of prolonged length of stay (pLOS) may help improve the medical efficiency and prognosis of PD-treated patients. If timely clinical interventions are not provided, patients at a high risk of pLOS may face a poor prognosis and high medical expenses, which will also be a burden on hospitals. Therefore, physicians need an effective pLOS prediction model for PD-treated patients.

**Objective:**

This study aimed to develop an optimal data-driven model for predicting the pLOS risk of PD-treated patients using basic admission data.

**Methods:**

Patient data collected using the Hospital Quality Monitoring System (HQMS) in China were used to develop pLOS prediction models. A stacking model was constructed with support vector machine, random forest (RF), and K-nearest neighbor algorithms as its base models and traditional logistic regression (LR) as its meta-model. The meta-model used the outputs of all 3 base models as input and generated the output of the stacking model. Another LR-based pLOS prediction model was built as the benchmark model. The prediction performance of the stacking model was compared with that of its base models and the benchmark model. Five-fold cross-validation was employed to develop and validate the models. Performance measures included the Brier score, area under the receiver operating characteristic curve (AUROC), estimated calibration index (ECI), accuracy, sensitivity, specificity, and geometric mean (Gm). In addition, a calibration plot was employed to visually demonstrate the calibration power of each model.

**Results:**

The final cohort extracted from the HQMS database consisted of 23,992 eligible PD-treated patients, among whom 30.3% had a pLOS (ie, longer than the average LOS, which was 16 days in our study). Among the models, the stacking model achieved the best calibration (ECI 8.691), balanced accuracy (Gm 0.690), accuracy (0.695), and specificity (0.701). Meanwhile, the stacking and RF models had the best overall performance (Brier score 0.174 for both) and discrimination (AUROC 0.757 for the stacking model and 0.756 for the RF model). Compared with the benchmark LR model, the stacking model was superior in all performance measures except sensitivity, but there was no significant difference in sensitivity between the 2 models. The 2-sided *t* tests revealed significant performance differences between the stacking and LR models in overall performance, discrimination, calibration, balanced accuracy, and accuracy.

**Conclusions:**

This study is the first to develop data-driven pLOS prediction models for PD-treated patients using basic admission data from a national database. The results indicate the feasibility of utilizing a stacking-based pLOS prediction model for PD-treated patients. The pLOS prediction tools developed in this study have the potential to assist clinicians in identifying patients at a high risk of pLOS and to allocate resources optimally for PD-treated patients.

## Introduction

Over the past 30 years, the United States Renal Data System has reported a rapid increase in the incidence of end-stage kidney disease (ESKD) [1]. The increasing number of patients with ESKD treated with kidney replacement therapy—including hemodialysis, peritoneal dialysis (PD), and renal transplantation—has put a large burden on the health care system. Approximately 2.6 million people worldwide received kidney replacement therapy in 2010 [2], and the prevalence of ESKD in China was 237.3 cases per million population in 2012 [3]. In 2015, the average inpatient expenditure for patients with ESKD in China was approximately ¥24,800 (US $3793) [4], and the total inpatient expenditure for patients with ESKD in China was in excess of ¥6.75 billion (US $1.03 billion). In 2016, the average expenditure on patients with ESKD in the United States was estimated to be US $50 billion, one-third of which was attributed to hospitalization costs [1]. Hospitalization remains a critical outcome for patients with ESKD, and the risk of hospitalization in patients undergoing dialysis is triple that of patients without ESKD [5]. In-hospital length of stay (LOS) is a key indicator of the efficiency of inpatient management. Prolonged LOS (pLOS) is associated not only with high resource consumption and medical expenses [6,7] but also with a high risk of complications [8]. Much attention has been given to reducing hospitalization costs [9-15], but few studies have focused on preventing pLOS for PD-treated patients. The increasing number of PD-treated patients and their consistently high hospital admission rate have placed a large burden on the health care system. An accurate pLOS prediction model can assist physicians to risk-stratify patients and optimally allocate health care resources [7,16]. Early clinical interventions and optimal management of patients at a high risk of pLOS may help reduce hospitalization expenses and improve prognosis for PD-treated patients [7,8,17]. If timely clinical interventions are not provided, patients at a high risk of pLOS may face poor prognosis and high medical expenses, which will also burden hospitals [18].

Given the increasing number of patients undergoing dialysis and the importance of optimal resource allocation, physicians need an effective LOS prediction model. However, no well-developed LOS prediction models for patients undergoing dialysis can be found in the literature. Some other risk-stratification models for patients undergoing dialysis use mortality [19-21] or cardiovascular events [22] as the end point. Wagner et al [20] used a nationwide, multicenter, prospective cohort study in the United Kingdom (the UK Renal Registry) as a data source to develop a Cox proportional hazards model for predicting long-term mortality in incident dialysis patients. They found that using basic patient characteristics, comorbid conditions, and laboratory variables to predict the 3-year mortality of incident dialysis patients had sufficient accuracy. Quinn et al [21] used a Canadian administrative health database to develop a prognostic index for 1-year mortality in patients undergoing dialysis by combining logistic regression (LR) with different variable selection methods. Matsubara et al [22] used data from the Japan Dialysis Outcomes and Practice Patterns Study to develop an LR model for predicting the incidence of cardiovascular events among patients undergoing hemodialysis. However, few models use LOS as the prediction outcome.

Meanwhile, a number of studies have explored the factors affecting the LOS of patients undergoing dialysis. Allon et al [23] explored the association of hospitalization outcomes with clinical factors and laboratory parameters in patients undergoing hemodialysis and found that infection-related hospitalization was associated with pLOS. Kshirsagar et al [24] compared the LOS of hemodialysis patients receiving care from nephrologists and internists and found that the LOS was significantly shorter for patients under the care of nephrologists than for patients under the care of internists. Rocco et al [25] studied the risk factors for hospitalization in patients receiving chronic dialysis and confirmed that the risk factors for LOS were similar to those for mortality. Other factors affecting the LOS of patients undergoing dialysis have also been explored, such as obesity [26], hemoglobin level [27], admission diagnosis [28], and comorbidities [23,29]. However, no study has built an effective model for pLOS prediction in patients undergoing dialysis.

With the exponential increase in the amount of health care data, machine learning algorithms have gained special attention for their capabilities of handling high-dimension and large-scale data. Some machine learning–based LOS prediction models have been developed for patients with other diseases. The prediction outcome of existing LOS prediction models could be classified into 2 types: (1) numeric LOS and (2) binary outcome (ie, having a pLOS or not). Moran et al [30] constructed a numeric LOS prediction model for patients in the intensive care unit (ICU) by using a traditional linear regression model. Their results suggested that their LOS prediction model performed well in predicting the average LOS of patients in the ICU but showed limited performance in predicting the LOS of individual patients. Yang et al [31] developed a numeric LOS prediction model based on the support vector machine (SVM) algorithm for burn patients at different stages and compared its prediction performance with that of the traditional linear regression model. They found that although the SVM model was more effective than the linear regression model in LOS prediction for burn patients, it yielded a high mean relative error of 43.9%. LaFaro et al [32] developed a numeric LOS prediction model based on the artificial neural network (ANN) algorithm for patients in the ICU after cardiac surgery. Their results also suggested that the ANN-based LOS prediction model outperformed the traditional linear regression model (R^2^: 0.410 vs 0.200; R^2^ measures the goodness of fit of the corresponding model), but the prediction performance of the ANN-based model was still limited. However, if patients are classified into 2 groups (ie, with and without pLOS), the difference in LOS patterns between patients in the 2 groups could be more obvious and easily discovered, and this classification helps identify typical LOS patterns and improve the performance of LOS prediction models [33]. In the literature, the LOS prediction models with binary outcomes achieved good performance. Ma et al [34] developed a personalized pLOS prediction model for patients in the ICU by combining just-in-time learning and one-class extreme learning machine algorithms and found that the model achieved superior performance to the traditional binary classification algorithms. Chuang et al [35] compared the performance of various supervised learning approaches with an LR model in pLOS prediction for general surgery patients and the results showed that the random forest (RF) model outperformed the LR model. Morton et al [36] used 5 machine learning algorithms to predict the pLOS of hospitalized patients with diabetes and found that the SVM model demonstrated the best prediction performance, followed closely by the RF model. However, LOS prediction models based on machine learning technologies for PD-treated patients remain to be developed.

Stacked generalization, or stacking, is a general ensemble method that combines different types of machine learning models (“base models”) through an aggregation model (“meta-model”) to maximize the prediction performance [37]. Several studies [38,39] have found that ensemble learning methods can produce a better or equal predictive performance than their component parts. Lertampaiporn et al [38] developed a heterogeneous ensemble model for microRNA precursor classification through a voting system. Their results showed that the ensemble method produced a more reliable prediction than its base classifiers. Wang et al [39] used the stacking algorithm to predict membrane protein types, and the ensemble model yielded a better overall performance than its base models. Phan et al [40] developed a stacking model to predict cancer survival and reported that this model outperformed the majority-vote model. An ensemble of various machine learning models could help reduce the bias in a single machine learning algorithm to provide a much better prediction performance than single models.

This study aimed to develop an optimal data-driven pLOS prediction model for PD-treated patients by using basic admission data from a national database. A pLOS prediction model was constructed for PD-treated patients by using the stacking method, and the Hospital Quality Monitoring System (HQMS) database in China was used for model development. An LR-based pLOS prediction model was built and considered as the benchmark model. The RF, SVM, and K-nearest neighbor (KNN) algorithms were employed as the base models because of their superior performance in constructing ensemble models [38,41], and the LR model was used as the meta-model for constructing the stacking model.

## Methods

### Data Set and Subjects

In this study, the HQMS database—a mandatory, patient-level national database in China—was used for data extraction and model development. The HQMS database is a large database consisting of standardized electronic inpatient discharge records, including 878 Class 3 hospitals in China [42]. The standardized electronic inpatient discharge record is a national standard medical record with a stringent standard format across different hospitals in China. The standardized electronic inpatient discharge records of patients must be filled in by clinicians who have the most comprehensive understanding of the patients’ medical conditions to ensure their validity. Strict automated data quality control was performed on the HQMS data reporting system. The completeness, accuracy, and consistency of data were assessed at the time of data submission to the HQMS. Patient demographic characteristics, clinical diagnoses, medical procedures, pathology diagnoses, and medical expenditures were included in the HQMS database.

This study was reviewed and approved by the Ethics Committee of Peking University First Hospital (2015-928). The HQMS data set used in this study spans from 2013 to 2015.

Patient records of individuals who met the following criteria were extracted from the HQMS data set: (1) aged between 18 and 100 years, and (2) treated with PD. Exclusion criteria were as follows: (1) diagnosed with acute kidney injury or kidney transplantation, and (2) died in the hospital. For patients readmitted on the same day as hospital discharge, we recalculated their LOS by merging the back-to-back admission records. The PD-treated patients were identified through admission and discharge diagnoses or in-hospital medical operations by using the International Statistical Classification of Diseases, Tenth Revision (ICD-10) codes (Multimedia Appendix 1). For PD-treated patients with several discontinuous hospitalizations, we randomly selected one record for each patient to ensure that all observations were independent and that PD-treated patients with varying severities were included for model development.

### Outcome and Predictor Variables

The prediction outcome of this study was binary (ie, having a pLOS or not). LOS was defined as the period from admission to discharge. pLOS was defined as an LOS longer than the average LOS, which is 16 days for patients with ESKD in China [43]. Patients with pLOS may have serious medical situations and thus need a longer hospital stay. We adopted this pLOS definition in our study by referring to existing studies [44-46] and consulting with experienced clinicians. The pLOS prediction models developed in our study aimed to assist physicians in identifying patients at a high pLOS risk and thus to provide early and timely interventions for these high-risk patients.

Predictor variables were determined on the basis of prior studies [23,24,28,29] and variable availability on admission. Variables used as predictor variables for model development in this study included age, sex, nationality, reason for admission, specific causes of chronic kidney disease (CKD), comorbidities, admission type, number of hospitalizations within 6 months, number of emergency admissions within 6 months, admission department, planned admission or not, admission day of the week, admitted in the same hospital as last admission or not, place of residence, and insurance type. The reason for admission, specific causes of CKD, and comorbidities were extracted using ICD-10 codes. The categories of reasons for admission and comorbidities were determined after consultation with experienced clinicians. Limited by the available data set, the number of hospitalizations within 6 months and number of emergency admissions within 6 months were calculated on the basis of the data collected from Class 3 hospitals.

### Model Development

#### RF Model

RF is a supervised ensemble learning algorithm consisting of a collection of tree-structured classifiers [47]. RF models work by generating a multitude of decision trees independently and then synthesizing the individual predictions of all trees through a voting system. Each tree in an RF model is built using a bootstrap sample of the training data set. Assuming that *M* predictor variables are included for model development, *F* of all *M* input variables are randomly selected for each node, and the split of each node is performed according to the minimal impurity principle. For each tree, a variable that was used for tree growth in the previous nodes will no longer be used in later splitting. In decision tree induction, the Gini index is a general impurity measure used to determine the splitting variables. If a data set *D* contains samples with *J* classes, the Gini index of data set *D*—Gini(*D*)—is defined as follows [48]:

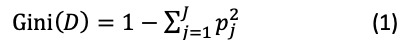



where *p_j_* is the frequency of the *j*th class in *D*. At each node, if a variable can split the parent data set *D* into 2 child data sets, *D*_1_ and *D*_2_, the decrease in the Gini index, *S*, for this variable is defined by the following:

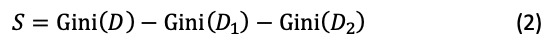



The variable with a maximal decrease in the Gini index will be used for splitting at this node.

In an RF model, to classify a new case, each tree in the forest model gives a classification result for the new case as a vote, and the majority vote is declared as the final classification of the model. Twice randomization in an RF model, which involves randomly selecting training data samples and randomly selecting the attributes for each tree growth, provides the model with a strong capability of handling high-dimensional data together with a stable generalization error [49].

We used the RandomForestClassifier package in Python to construct the RF model in this study. A set of optimal parameters of the RF model was found using grid search, which is an exhaustive searching method using a manually specified subset of hyperparameter space to find the optimal parameters of a learning algorithm [50]. The RF model obtained in this study had the following parameters: the number of decision trees was 300, the number of variables (*F*) selected at each node was 10, and the maximal depth of each decision tree was 28.

#### SVM Model

SVMs have been used frequently in various classification problems because of their remarkably robust performance in handling noisy and nonlinearly classified data [51]. If the data set is not linearly separable, a mapping function will be used in the SVM to map the data set into a high-dimensional space. An SVM tries to find an optimal separating hyperplane (ie, the maximum-margin hyperplane) in the high-dimensional space to make a classification. Assuming that a training data set, *D,* consists of *N* labeled cases, 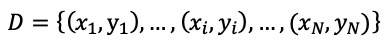, where *x_i_* represents the *i*th feature vector and *y_i_* is the label of the *i*th case. A mapping function, ø (*x*), will map the data set from the original space into a high-dimensional space. In the transformed high-dimensional space, the separating hyperplane [52] is defined as follows:

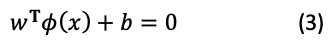



where is a normal vector determining the direction, and *b* is the bias. The training cases with minimum margins from the hyperplane are called support vectors. A support vector (*x_j_*, *y_j_*) satisfies:

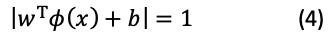



In the high-dimensional space, the margin *M* between the support vector and the hyperplane is defined as

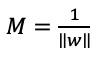



The hyperplane that makes the margin *M* maximum is the optimal separating hyperplane (ie, maximum-margin hyperplane). In the process of finding the optimal separating hyperplane, a kernel function is usually used to deal with the high computational cost. Commonly used kernel functions include the polynomial kernel, the linear kernel, the exponential kernel, and the radial basis function kernel.

We used the svm package in Python to construct the SVM model, and the optimal parameters of our SVM model were found using grid search. The SVM model obtained in this study had the following parameters: the kernel function was polynomial kernel, the degree of the polynomial kernel function was 2, and the penalty parameter C was 0.01.

#### KNN Model

KNN is a type of instance-based learning method that makes predictions based on a small number of cases that are very similar to the target observation [53]. Specifically, given a new case (*x_new_*), we can find the K closest training cases, 
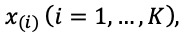
 sorted by the distance to *x_new_*, and then classify *x_new_* using majority voting among the K neighbors. A commonly used distance metric in the KNN algorithm is the Euclidean distance. Assuming the presence of case 
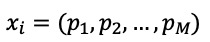
 and 
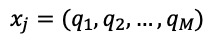
, we define the Euclidean distance from *x_i_* to *x_j_* as

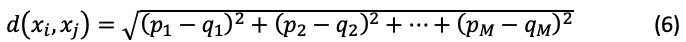



where 
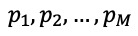
 and 
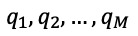
 denote the values of *M* input predictor variables of the 2 cases. Typically, we first normalize all the values of the variables to the range of (0,1) because different variables could be measured in different units. The KNN algorithm yields convincing results in handling various classification problems in medicine [54-56]. The model is effective on data sets where samples of 1 class have many possible patterns and the decision boundary is nonlinear [57]. The most important parameter in the KNN model is the number of neighbors, which must be selected with care. In this study, we used the KNeighborsClassifier package in Python to construct the KNN model. The optimal parameter *K* was found using grid search, and the KNN model with optimal performance was obtained with the parameter *K*=130.

### Stacked Generalization

Stacked generalization, or stacking, is an ensemble model that can combine the predictions of several primary machine learning models [37]. There are 2 types of models in a stacking framework: several base models (level-0 models) and 1 meta-model (level-1 model). The meta-model is employed to combine the base models. In general, a stacking framework can obtain a more accurate prediction result than any single base model. Different models may complement each other, and the meta-algorithm can combine the advantages of these base models.

The stacking model is trained as follows. Given a data set 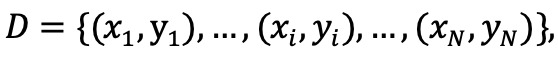 we define *D_k_* and *D_–k_* = *D* – *D_k_* as the training and test data sets, respectively, in the *k*th round of model training. We assume that the stacking model has *J* base models (*Model*_1_, *Model*_2_, ... , *Model_j_*, ... *Model_J_*) and that each base model is trained using *D_k_*. Let 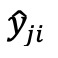 denote the prediction outcome produced by *Model_j_* for training case (*x_i_*, *y_i_*). The outputs of all *J* base models are assembled as the input of the meta-model. Let 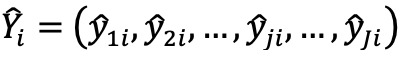 denote the set of outputs produced by all of the *J* base models for (*x_i_*, *y_i_*). The meta-model is then trained using data set 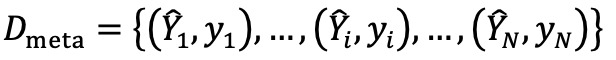.

**Figure 1:**
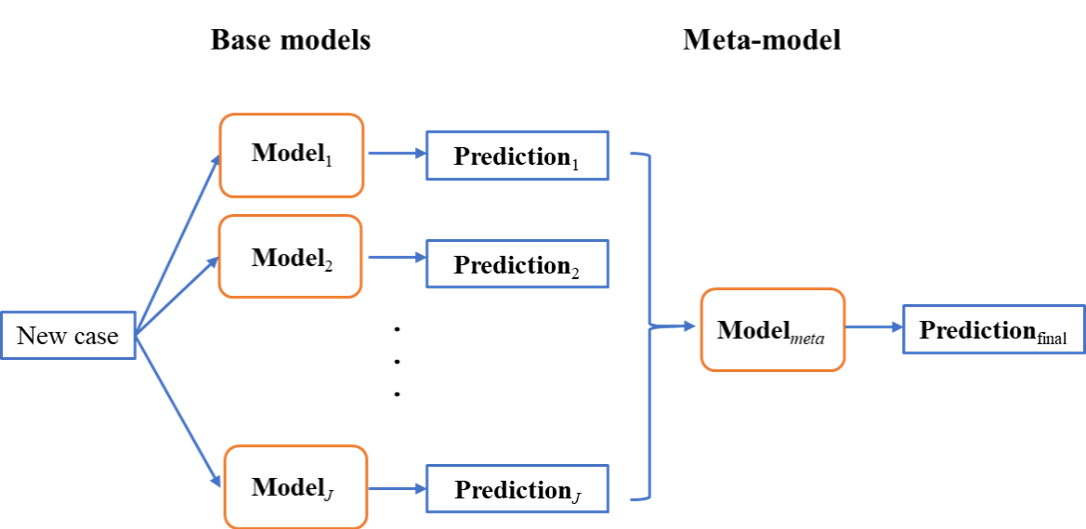
Stacked generalization, where Predictionj denotes the prediction outcome produced by the model (Modelj) for a new case.

For a new input case, the output of the meta-model is the final prediction outcome produced by the stacking model for the case. How the base models are assembled in the stacking method and how the prediction outcome for a new input case is generated by the stacking model are shown in Figure 1.

Given that the level-0 base models have already completed most of the prediction work, the level-1 meta-model could be rather simple [58]. The LR model is commonly used as the meta-model. Existing studies [37,59] suggested that increasing diversity of the base models could help improve the performance of the stacking model. In this study, the RF, SVM, and KNN models were employed as the base models and the LR model was used as the meta-model.

### Statistical Analysis

Two-sided *t* tests and chi-square tests were used for comparisons of patient demographics. In model development and comparisons, we employed 5-fold cross-validation. In performance comparisons, the Brier score [60], area under the receiver operating characteristic curve (AUROC) [60], estimated calibration index (ECI) [61], accuracy, sensitivity, specificity, and geometric mean (Gm) [62] were employed as performance measures. Considering that other performance metrics, such as positive and negative predictive values and likelihood ratios, can be calculated from sensitivity and specificity, we did not employ them in performance comparisons. Brier score is an overall performance measure, with a lower Brier score suggesting a superior overall prediction performance. AUROC measures the discrimination power of a prediction model, representing the ability to distinguish positive samples from negative samples. ECI measures the calibration power of a model, representing the average difference between the predicted probabilities of individual patients and the observed probability in that patient population. ECI ranges between 0 and 100, with a lower ECI suggesting a stronger calibration power of the corresponding model. Gm is considered a balanced accuracy measure because it incorporates sensitivity and specificity, and it is defined as follows:

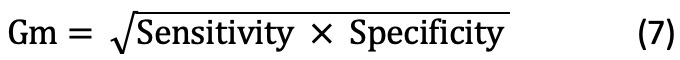



Gm measures the balance of the classification performance for the majority and minority classes. The optimal cutoff value for each model was obtained according to its corresponding receiver operating characteristic curve, and then accuracy, sensitivity, specificity, and Gm were calculated. Performance differences between different models were assessed using 2-sided *t* tests. Furthermore, we used the calibration plot [60] to demonstrate the calibration power of each model in different patient groups with pLOS risk from low to high. In the calibration plot, patients were divided into 10 groups according to their predicted pLOS probabilities. The x-axis shows the observed pLOS probability of each patient group, and the y-axis shows the averaged predicted pLOS probability of each group. The ideal calibration curve for a perfect model is a diagonal, which suggests that the predicted probabilities are exactly consistent with the observed probabilities.

Statistical analysis and calculations were performed using Python 3. Less than 15% of records in the HQMS database had missing values for the nationality and admission type variables, and the missing values were considered as a special category in the analysis.

## Results

**Figure 2:**
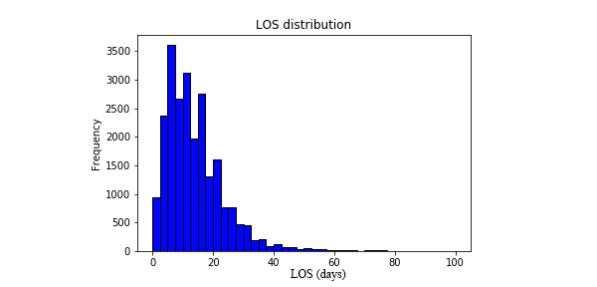
Histogram of length of stay (LOS) distribution of peritoneal dialysis–treated patients.

A total of 23,992 eligible patients receiving PD were included in our study, of whom 30.3% had a pLOS. Characteristics of the PD-treated patients are displayed in Table 1. The proportion of male patients was 55.6% (13,351/23,992), and the average age of all patients was 52.1 (SD 15.0) years. The 2-sided *t* tests showed that the differences in age, place of residence, and insurance type between PD-treated patients with a pLOS and those without a pLOS were statistically significant. The histogram of the LOS distribution of the PD-treated patients is displayed in Figure 2.

**Table 1:** Characteristics of peritoneal dialysis–treated patients in the study.

Characteristic				All patients		Patients with pLOS^a^		Patients without pLOS		*P* value
Number of patients (%)				23,992 (100)		7270 (30.3)		16,722 (69.7)		
Age (years), mean (SD)				52.1 (15.0)		53.6 (15.4)		51.5 (14.8)		<.001
**Sex, n (%)**										.63
		Female	10,641 (44.4)		3242 (44.6)		7399 (44.2)			
		Male	13,351 (55.6)		4028 (55.4)		9323 (55.8)			
**Place of residence, n (%)**										<.001
		East China	9425 (39.3)		2565 (35.3)		6860 (41.0)			
		North China	2318 (9.7)		902 (12.4)		1416 (8.5)			
		Central China	3416 (14.2)		1157 (15.9)		2259 (13.5)			
		South China	3978 (16.6)		1261 (17.3)		2717 (16.2)			
		Southwest China	2933 (12.2)		849 (11.7)		2084 (12.5)			
		Northwest China	1067 (4.4)		225 (3.1)		842 (5.0)			
		Northeast China	855 (3.6)		311 (4.3)		544 (3.3)			
**Insurance, n (%)**										.005
		UEBMI^b^	9100 (37.9)		2714 (37.3)		6386 (38.2)			
		URBMI^c^	2192 (9.1)		705 (9.7)		1487 (8.9)			
		NRCMS^d^	6082 (25.4)		1931 (26.6)		4151 (24.8)			
		Free medical care	334 (1.4)		101 (1.4)		233 (1.4)			
		Self-paid treatment	3493 (14.6)		997 (13.7)		2496 (14.9)			
		Other	2791 (11.6)		822 (11.3)		1969 (11.8)			

^a^pLOS: prolonged length of stay.

^b^UEBMI: urban employee basic medical insurance.

^c^URBMI: urban resident basic medical insurance.

^d^NRCMS: new rural cooperative medical system.

A comparison of the prediction performance of the stacking model, its 3 base models, and the benchmark LR model in terms of the Brier score, AUROC, ECI, Gm, accuracy, sensitivity, and specificity is shown in Table 2. Among these models, the stacking model achieved the best calibration (ECI 8.691), balanced accuracy (Gm 0.690), accuracy (0.695), and specificity (0.701). Meanwhile, the stacking and RF models had the best overall performance (Brier score 0.174 for both) and discrimination (AUROC 0.757 for the stacking model and 0.756 for the RF model). Compared with the benchmark LR model, the stacking model was superior in all performance measures except sensitivity, but there was no significant difference in sensitivity between the 2 models. The 2-sided *t* tests revealed significant performance differences between the stacking and LR models in overall performance, discrimination, calibration, balanced accuracy, and accuracy.

**Table 2:** Prediction performance of the 5 models.

Model	Brier score	AUROC^a^ (95% CI)	ECI^b^	Gm^c^	Accuracy	Sensitivity	Specificity
LR^d^	0.178	0.742 (0.731-0.753)	8.911	0.677	0.675	0.683	0.671
KNN^e^	0.188*	0.721 (0.703-0.740)*	9.386*	0.661*	0.666	0.666	0.657
SVM^f^	0.187*	0.730 (0.720-0.739)*	9.342*	0.673	0.680	0.656	0.690
RF^g^	0.174*	0.756 (0.748-0.765)*	8.722*	0.689*	0.691*	0.686	0.693
Stacking	0.174*	0.757 (0.748-0.765)*	8.691*	0.690*	0.695*	0.680	0.701

^a^AUROC: area under the receiver operating characteristic curve.

^b^ECI: estimated calibration index.

^c^Gm: geometric mean.

^d^LR: logistic regression.

^e^KNN: K-nearest neighbor.

^f^SVM: support vector machine.

^g^RF: random forest.

*P<.05 in 2-sided *t* test when compared with the LR model.

**Figure 3:**
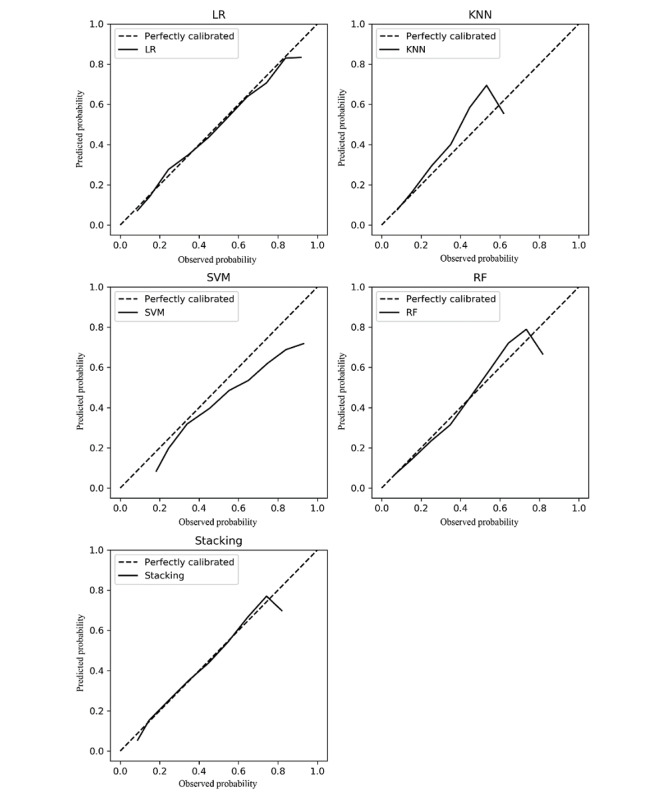
Calibration plots of the 5 models. KNN: K-nearest neighbor; LR: logistic regression; RF: random forest; SVM: support vector machine.

Figure 3 demonstrates the calibration plots of the 5 models. The calibration curve of the stacking model was the optimal fitting curve among the 5 models. The SVM model underestimated the pLOS probabilities for most patients, whereas the KNN model overestimated the pLOS probabilities for most patients. The RF model underestimated the pLOS probabilities for most patients at low risk and overestimated the probabilities for most patients at high risk.

## Discussion

### Principal Findings

The main objective of this study was to develop an optimal data-driven model for predicting the pLOS risk of PD-treated patients using basic admission data. To the best of our knowledge, this study is the first to develop such pLOS prediction models for PD-treated patients by using data from a national database. Our study constructed a pLOS prediction model for PD-treated patients based on a stacking method with KNN, SVM, and RF as its base models and LR as its meta-model. The prediction performance of the stacking model was compared with those of a benchmark LR model and its 3 base models. A pragmatic pLOS prediction model for PD-treated patients would be useful in family consultation and has the potential to assist physicians in making optimal clinical decisions. Considering that medical expenses are highly associated with LOS [6,7], the pLOS prediction model could help estimate the medical expenses for PD-treated patients. The degree of satisfaction may increase if patients and their families know more about their LOS and medical expenses on hospital admission. In addition, the pLOS prediction models could be integrated into hospital information systems, providing physicians with real-time suggestions about the LOS of patients and helping physicians to identify PD-treated patients at a high risk of pLOS and give timely individualized intervention.

In this study, the RF, SVM, and KNN models were employed as base models for stacking because they have different learning mechanisms and have advantages in different aspects. RF is an ensemble learning algorithm consisting of a collection of tree-structured classifiers. The twice randomization in an RF model provides the model with a strong capability of handling high-dimensional data together with a stable generalizability [49]. However, RF models are sensitive to noise data. SVM models make classifications by mapping data into a high-dimensional space and finding an optimal separating hyperplane in the high-dimensional space. SVM models show remarkably robust performance in handling noisy and nonlinearly classified data but have limitations in handling high-dimensional data [51]. KNN is an instance-based learning method that makes predictions depending on a small number of cases that are strongly similar to the target observation. KNNs are effective on nonlinearly separable data sets and data sets where samples of one class have different patterns [57]. KNNs are insensitive to noise data but have limited accuracy in unbalanced data. In addition, an existing study [38] showed that the ensemble of the 3 models demonstrated superior prediction performance in dealing with classification problems. Moreover, the literature states that the 3 classifiers are suitable for pLOS prediction problems. All 3 classifiers have shown superior performance in predicting pLOS for patients. Chuang et al [35] employed the SVM and RF models for pLOS prediction in patients who underwent general surgery, and both models achieved a high AUROC. Steele and Thompson [63] developed a KNN-based pLOS prediction model for general patients and achieved an AUROC of 0.847. KNN was included as the base model in our study because it has shown superior performance in pLOS prediction in existing studies [63,64]. Given that its learning mechanism is different from the learning mechanisms of the 2 other base models (SVM and RF), KNN was expected to improve the prediction performance of the stacking model in dealing with data sets with various characteristics [37,59]. We also attempted to construct stacking models with combinations of any 2 base models of RF, SVM, and KNN. We found that the stacking model with SVM and KNN as its base models had the worst performance, while the stacking model with 3 base models and the stacking models with the other 2 combinations (SVM and RF, and KNN and RF) had similar overall performances. Considering the diversity and respective advantages of the base models, and the generalizability of the stacking model in dealing with data sets with different characteristics, we selected the stacking model with 3 base models.

The performance comparison results showed that the stacking model was the best among the 5 models in terms of overall performance (Brier score), discrimination (AUROC), calibration (ECI), balanced accuracy (Gm), accuracy, and specificity. The RF model showed the best prediction performance among the 3 base models, and it had a similar overall performance and discrimination power as the stacking model. The good prediction performance of the stacking and RF models may be due to the fact that both models are ensemble learning models. Our study results are consistent with previous studies showing that the ensemble model is almost always superior to single learning models [38,39]. A stacking model can exploit its base models by combining the output of each model via a meta-model, thus reducing the bias that tends to occur with a single classifier. An RF model can exploit its base tree models by combining the output of each model via a voting system. The stacking model was slightly superior to the RF model in most performance measures for 2 possible reasons. First, the prediction performance of a stacking model is usually similar to its best base model [40,41]. Second, compared with an RF model, a stacking model has more diverse base models that can complement each other.

The calibration curves of the 5 models further suggest that the stacking model had the optimal calibration power in different patient groups. ECI measures the overall calibration power of a model, whereas the calibration curve visually shows the calibration power of a model in patient groups with pLOS risk from low to high. The ECI and calibration curve demonstrated that the stacking model had superior calibration power. The calibration curve showed that the averaged predicted pLOS probability of the stacking model had high consistency with the observed outcome across different pLOS risk groups. Meanwhile, the calibration curve showed that the RF model underestimated the pLOS probabilities of most patients at low risk and overestimated the probabilities of most patients at high risk. This feature can help the RF model expand the difference of predicted probabilities between patients with different pLOS risks and thus discriminate the patients at a high pLOS risk from those at a low risk. This probably explained why the RF model showed similar discrimination but worse calibration power than the stacking model.

We also attempted to develop numeric LOS prediction models for PD-treated patients, but the corresponding prediction performance of the models was limited, which was similar to that of existing numeric LOS prediction models. Numeric LOS prediction models focused on mining different LOS patterns for patients with different LOSs (even 1 day apart), but the difference in LOS patterns between patients with different LOSs, especially those LOSs with 1 or 2 days apart, may be slight and was difficult to identify. The pLOS prediction models with binary outcomes had a much better performance.

Regarding data exclusion, the PD-treated patients who died in the hospital were excluded in our study because the LOS pattern of the decedents might be different from that of patients who survived in the hospital [65,66]. Based on our consultations with experienced clinicians, we knew that there was uncertainty in the LOS pattern of patients who died in the hospital. Specifically, deceased patients could die quickly after hospital admission and have a short LOS or die after a long period of treatment and have a long LOS. In fact, the proportion of PD-treated patients who died in the hospital was only 0.8% in our study. Selection bias might have occurred when we excluded those PD-treated patients who died in the hospital, and the pLOS prediction model developed in our study may not apply to those patients who have a high risk of in-hospital mortality.

In our study, some PD-treated patients were hospitalized more than once; they can be classified into 2 types: (1) patients readmitted on the same day as discharge, and (2) patients with several discontinuous hospitalizations. Some hospitals in China may discharge patients with a potential pLOS first and then readmit them on the same day to reduce the average LOS, which is an important indicator in hospital evaluation. Therefore, for the PD-treated patients readmitted on the same day as discharge, we recalculated their actual LOS by merging the back-to-back admission records in this study. To deal with the situation of PD-treated patients with several discontinuous hospitalizations, we examined 2 approaches that were employed in the literature: (1) selecting the first hospitalization record, or (2) randomly selecting 1 record among multiple hospitalization records. Compared with the former approach, the latter approach may help include patients with varying severities [67]. Thus, we employed the second approach and randomly selected 1 record for each patient to ensure that all observations were independent and PD-treated patients with varying severities were included in model development.

### Definition of pLOS

In this study, pLOS was defined as an LOS longer than the average LOS by referring to existing studies [44-46] and consulting with experienced clinicians. In the literature, there is no consensus on the definition of pLOS for general patients or PD-treated patients. Existing studies have defined pLOS as an LOS longer than the average LOS [44-46], longer than the median LOS [68], or longer than a specific LOS according to experiences [69]. After consulting with experienced clinicians, we know that the average LOS is a more important metric for PD-treated patients, and it is also a more commonly used metric in assessing medical efficiency around the world. In addition, pLOS has been defined as an LOS longer than the average LOS in various medical fields by researchers from different countries [44-46]. Among the 3 cited references that defined pLOS as an LOS longer than the average LOS, one study [44] was of trauma patients in the United States, another study [45] was of critically ill patients in Switzerland, and the third study [46] was of surgery patients in China. Therefore, the definition of pLOS as longer than the average LOS may help our models achieve good generalizability to some extent.

### Diagnosis Codes

The use of diagnosis codes to identify patients with specific diseases may miss some target patients because clinicians tend to focus on the main diagnosis related to admission reasons and overlook the diagnosis of other diseases. To address this problem, we employed ICD-10 codes associated with all admission and discharge diagnoses and in-hospital medical operations to identify PD-treated patients. We also used ICD-10 codes associated with admission and discharge diagnoses to identify patients' comorbidities.

### Strengths and Limitations of the Study

This study has several strengths. First, a large nationwide database with a relatively representative population was used to derive the prediction models. Second, all of the predictor variables are available at admission, which ensures the feasibility of applying the developed models in clinical practice to assist clinical decision making. Third, 5-fold cross-validation was employed to achieve reliable performance results.

However, this study has some limitations. First, the models were derived from a nationwide data set in China. Some of the variables included in the models, such as nationality and insurance type, are region specific. The generalizability and validity of our prediction models need to be validated using a data set from different regions. Second, other potentially important variables, such as some laboratory markers, that reportedly affect LOS [27,70] were not available in the studied data set. Third, only patient data from Class 3 hospitals were included in the studied data set. Class 3 hospitals in China provide the best medical services for patients, and patients admitted to Class 3 hospitals in China may be suffering from serious diseases. Thus, our pLOS prediction models may not be applicable to the PD-treated patients in the primary or Class 2 hospitals in China, considering that patients admitted to those hospitals may have only minor or moderate diseases.

### Conclusion

This study was the first to develop data-driven automated pLOS prediction models for PD-treated patients using basic admission data from a national database. The results of our study indicate the feasibility of utilizing a stacking-based model for PD-treated patients. The developed pLOS prediction models have the potential to help clinicians identify PD-treated patients at a high risk of pLOS and then provide optimal patient management. The pLOS prediction tools developed in this study have the potential to assist clinicians in identifying patients at a high risk of pLOS and to allocate resources optimally for PD-treated patients. The generalizability and validity of the developed pLOS prediction models need to be externally validated, and the clinical utility of the models needs further validation before they are used in clinical practice. The pLOS prediction models developed in our study are purely theoretical so far, and we plan to integrate them into the information system of a pilot hospital for prospective validation.

## Appendix

Multimedia Appendix 1 International Statistical Classification of Diseases, Tenth Revision (ICD-10) codes for identifying peritoneal dialysis patients.

## Abbreviations

ANN: artificial neural network
AUROC: area under the receiver operating characteristic curve
CKD: chronic kidney disease
ECI: estimated calibration index
ESKD: end-stage kidney disease
Gm: geometric mean
HQMS: Hospital Quality Monitoring System
ICD-10: International Statistical Classification of Diseases, Tenth Revision
ICU: intensive care unit
KNN: K-nearest neighbor
LOS: length of stay
LR: logistic regression
PD: peritoneal dialysis
pLOS: prolonged length of stay
RF: random forest
SVM: support vector machine
