# Supplementary material for: Predicting Prolonged Length of Hospital Stay for Peritoneal Dialysis–Treated Patients Using Stacked Generalization: Model Development and Validation Study
Source: JMIR Med Inform. 2021 May 19;9(5):e17886. doi: 10.2196/17886 (PMC8173398; doi:10.2196/17886)
Supplement: Multimedia Appendix 1 [file medinform_v9i5e17886_app1.docx]

# Multimedia Appendix 1

Table A1. International Statistical Classification of Diseases, Tenth Revision (ICD-10) codes for identifying peritoneal dialysis patients

| **Diagnosis/Operation** | **Version** | **ICD-10 codes** |
| --- | --- | --- |
| Diagnosis | National | T85.609, T85.610, T85.611, T85.710, T85.711, T85.801, T85.901, Z49.201 |
|  | Beijing | T80.201, T85.602, Z49.201, Z99.202 |
|  | Clinical | T85.609, T85.610, T85.611, T85.700x103, T85.700x104, T85.710, T80.200x001, T85.711, T85.801, T85.901, Z49.201 |
| Operation | Beijing | 54.93002, 54.98001, 97.86003 |
|  | Clinical | Z45.800x007 |

Note: Three different ICD-10 code versions: National, Beijing and Clinical were used in the studied dataset.
